# A standard method to synthesize Ag, Ag/Ge, Ag/TiO_2_, SnO_2_, and Ag/SnO_2_ nanomaterials using the HVPG technique

**DOI:** 10.1016/j.mex.2019.11.025

**Published:** 2019-11-28

**Authors:** Muhammad Akhsin Muflikhun, Gil Nonato C. Santos

**Affiliations:** a Department of Mechanical and Industrial Engineering, Faculty of Engineering, Gadjah Mada University, Jl. Grafika No.2, Yogyakarta 55281, Indonesia; b Physics Department, De La Salle University, 2401 Taft Avenue, Manila, Philippines

**Keywords:** Standard method for synthesis nanomaterials using Horizontal Vapor Phase Growth (HVPG) technique, HVPG, Nanomaterials, Material phase change, Vacuum condition, Sealing

## Abstract

Nanotechnology is growing rapidly in the past few decades with the applications in several fields such as medicine, environment, energy, electronics, automotive, and aerospace. There are many methods used by researchers to synthesize nanomaterial. In this paper, Horizontal Vapor Phase Growth (HVPG) technique was successfully used to synthesize various nanomaterial and nanocomposite materials such as Ag, Ag/Ge, Ag/TiO_2_, SnO_2_, and Ag/SnO_2_. HVPG technique used a one-pot step to synthesize nanomaterials with 100 % purity of the results, affordable cost, and environmentally friendly. The method has two variables; growth temperature and curing time. Changing the variables create different shapes of nanomaterials. It also reported that the technique could be used to synthesize various nanomaterials consists of single or multi-material. This detailed method demonstrates the capability of the HVPG technique to synthesize nanomaterials, not only to grow the single shape of nanomaterials but also allow other nanomaterial shapes to grow in different parameter conditions.

• HVPG technique successfully used to synthesize various nanomaterials.
• Only 2 parameters used; curing time and growth temperature.
• Purity result (100 %) with no pollutant.

## Graphical abstract

Phase change from macro size to nano size in HVPG where the nanomaterial can be seen mostly in Zone 2 and 3.

**Figure:**
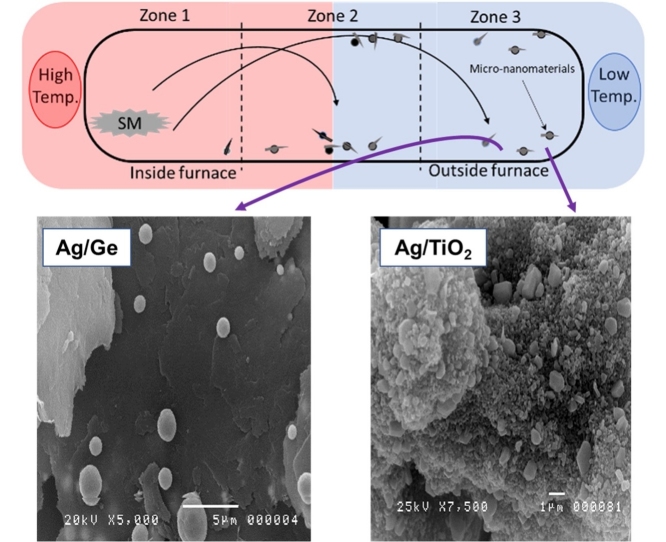

**Specification Table**

**Table:** 

Subject Area	Engineering
More specific subject area:	Synthesis nanomaterials
Method name:	Standard method for synthesis nanomaterials using Horizontal Vapor Phase Growth (HVPG) technique
Name and reference of original method:	[1] M.A. Muflikhun, M.C. Frommelt, M. Farman, A.Y. Chua, G.N.C. Santos, Structures, mechanical properties and antibacterial activity of Ag/TiO2 nanocomposite materials synthesized via HVPG technique for coating application, Heliyon. 5 (2019) 1–21. doi:https://doi.org/10.1016/j.heliyon.2019.e01475. [2] G. Santos, E. Tibayan, G. Castillon, E. Estacio, T. Furuya, A. Iwamae, K. Yamamoto, M. Tani, Tin Oxide-Silver Composite Nanomaterial Coating for UV Protection and Its Bactericidal Effect on Escherichia coli (E. coli), Coatings. 4 (2014) 320–328. doi:https://doi.org/10.3390/coatings4020320.
Resource availability:	All resource is listed in the methodsX paper

## Method details

### Overview

Nanomaterials that consist of Silver (Ag), Titanium dioxide (TiO_2_), and Tin dioxide (SnO_2_) were investigated by many researchers in the past decades. In general, synthesis of Ag nanomaterials was introduced by many researchers via three methods; chemical methods, physical methods, and biological methods. In each method, there are several routine processes that need to be followed to make Ag nanomaterials. They show that by using nanomaterials, many human problems in various aspects can be solved using a specific type of nanomaterials for example Drug delivery, anti-bacterial, bio sensor, protein detection, tissue engineering, and tumor destruction [[3], [4], [5], [6], [7], [8], [9]]. A study conducted by Wei et al. shows that Ag nanoparticles were effective to use in therapeutic applications for various diseases in the medical field [10]. Chen and Mao observed many methods that can be used to synthesize TiO_2_ [11]. They show 15 different methods that can be used with varying results. Furthermore, this study also describes the applications of TiO_2_ in the field of photocatalytic, sensing applications, and photovoltaic. In other hand, studied the advanced design, synthesis, and applications of SnO_2_ nanomaterials has been done by Wang and Rogach [12]. They show that SnO_2_ can be used in a wide range of applications such as gas sensors, lithium-ion batteries, and conductive film.

Although numerous methods were successfully proven to be used to synthesize nanomaterial such as Ag/TiO_2_ and Ag/SnO_2_, there are several reports that researchers concern about the safety and environment issue. Many methods show high result in fabricating nanomaterial with environmental issue, such as some synthesis methods were reported used catalyst and harmful material, for example, HCl that need higher standard and safety concern due to health issues and environmentally friendly issue [[13], [14], [15], [16]]. Moreover, studies also reported that in developing countries, the health risks of exposure unhealthy nanomaterials by direct contact still lack of understood. This issue was spread in large aspect since the regulation, safety, fabrication process, and proper knowledge is not satisfied with the standard procedure [17,18]. To deal with these issues, synthesis nanomaterial that have environmentally friendly, can deal with safety issues, and ensure to produce higher results is the need of the hour.

Upon assessment of previous work, there is no study on how to synthesize nanomaterial showing a detailed method that replicate due to the complexity during the synthesis process. Moreover, there is no study that show detail method to synthesis nanomaterial that have many advantages for instance cheaper (using quartz tube), have safety concern by removing any catalyst and any harm materials, also one pot technique. The purpose of this study is to make a bridge between synthesis Ag, TiO_2_, and SnO_2_ in a detailed sequence and using the single pot synthesis method. The author aims to give the detail process on how to synthesis various nanomaterials with the prominent result using the so-called Horizontal Vapor Phase growth (HVPG) technique.

**Fig. 1:**
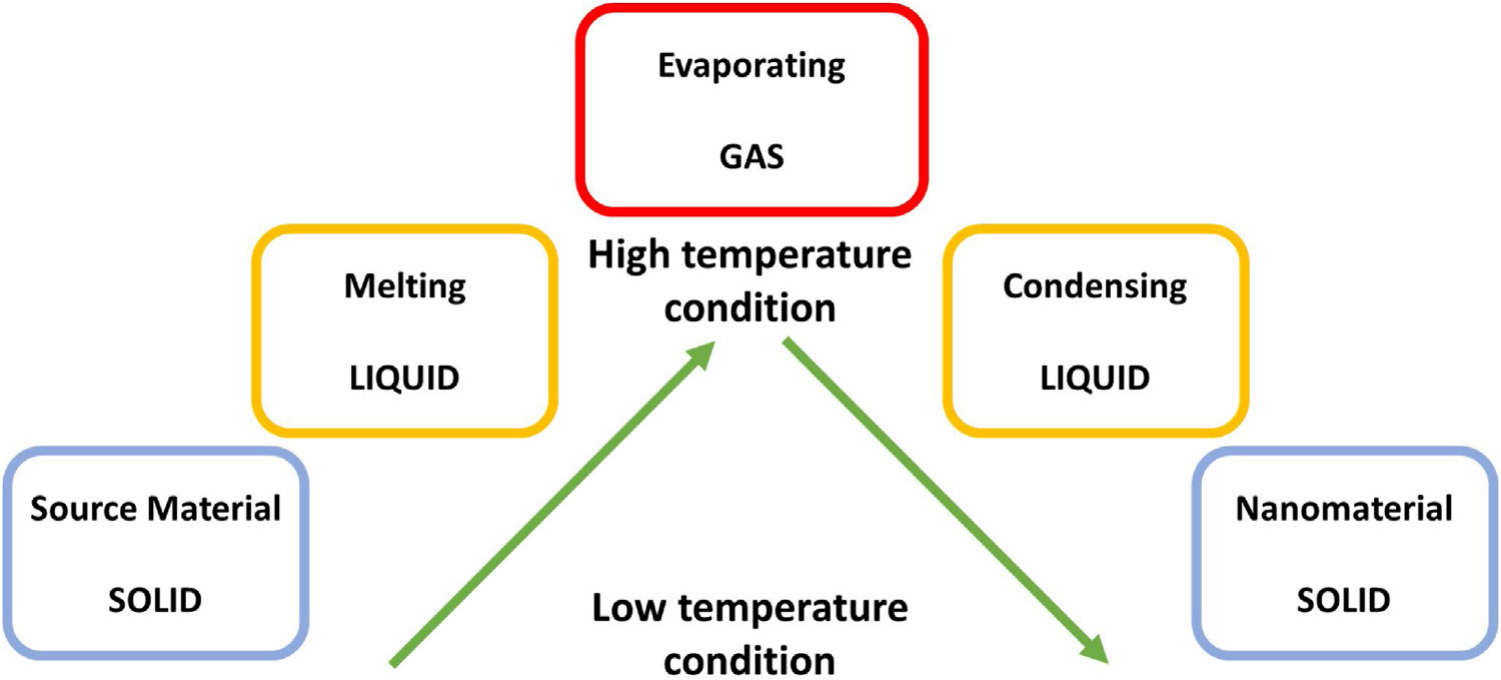
Schematic material phase change.

HVPG technique is based on the material phase change at high temperature as it reaches its melting point properties. The material phase change basically starts from a solid source material with macro sizes, transitioning to liquid form when the material reaches its melting point and then evaporating due to a temperature difference in different zones. When the material evaporated and then reaches cooler region, it starts condensed and changed the form to solid after completely adhered to the inside tube surface. For the detail, Fig. 1 shows the schematic material phase change that occurred during the HVPG technique.

**Fig. 2:**
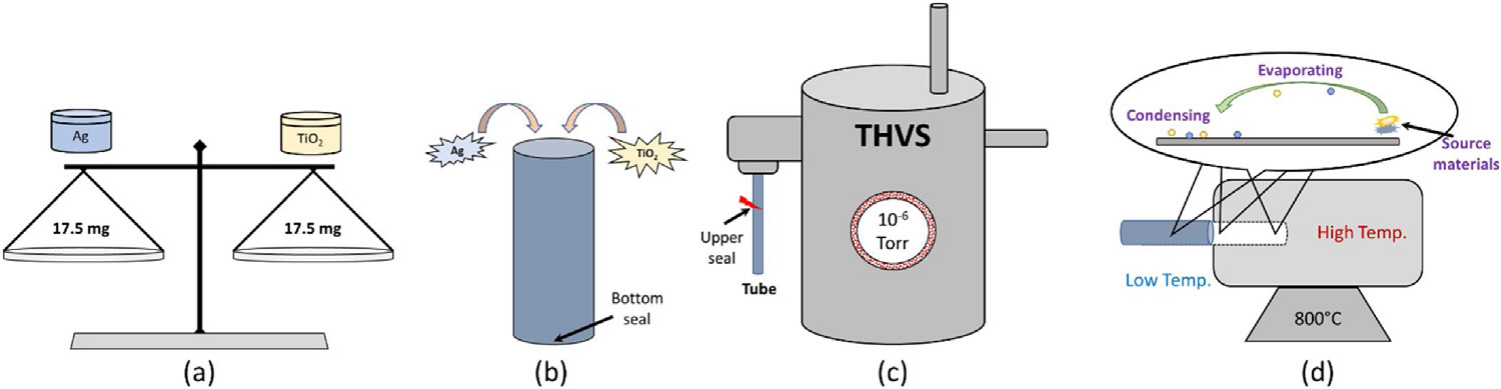
HVPG technique sequences. (a) measure source materials, (b) seal one-end of the tube with the inserted powder material (c) sealing process using a blowtorch at the other end of the tube with a vacuum pressure of 10^−6^ Torr, and (d) nanomaterial growth process.

In general, synthesis of nanomaterial using the HVPG technique can be divided into three parts; Preparation, Sealing, and Curing. The following sections describe in detail about each step. Fig. 2 illustrates the procedural steps to synthesize nanomaterial using Ag and TiO_2_ as a representative sample. The process started with measuring powder materials with a fixed amount of 35 mg (Fig. 2 (a)). The material is then poured into the quartz tube which is sealed on one-end (Fig. 2 (b)). The tube was then attached to a Thermionic High Vacuum System (THVS) and was evacauted with a vacuum pressure of 10^−6^ Torr (Fig. 2 (c)). The tube was sealed and was placed horizontally in a tube furnace to grow the nanomaterials (Fig. 2 (d)) with a growth temperature of 800 °C.

### Preparation

**Fig. 3:**
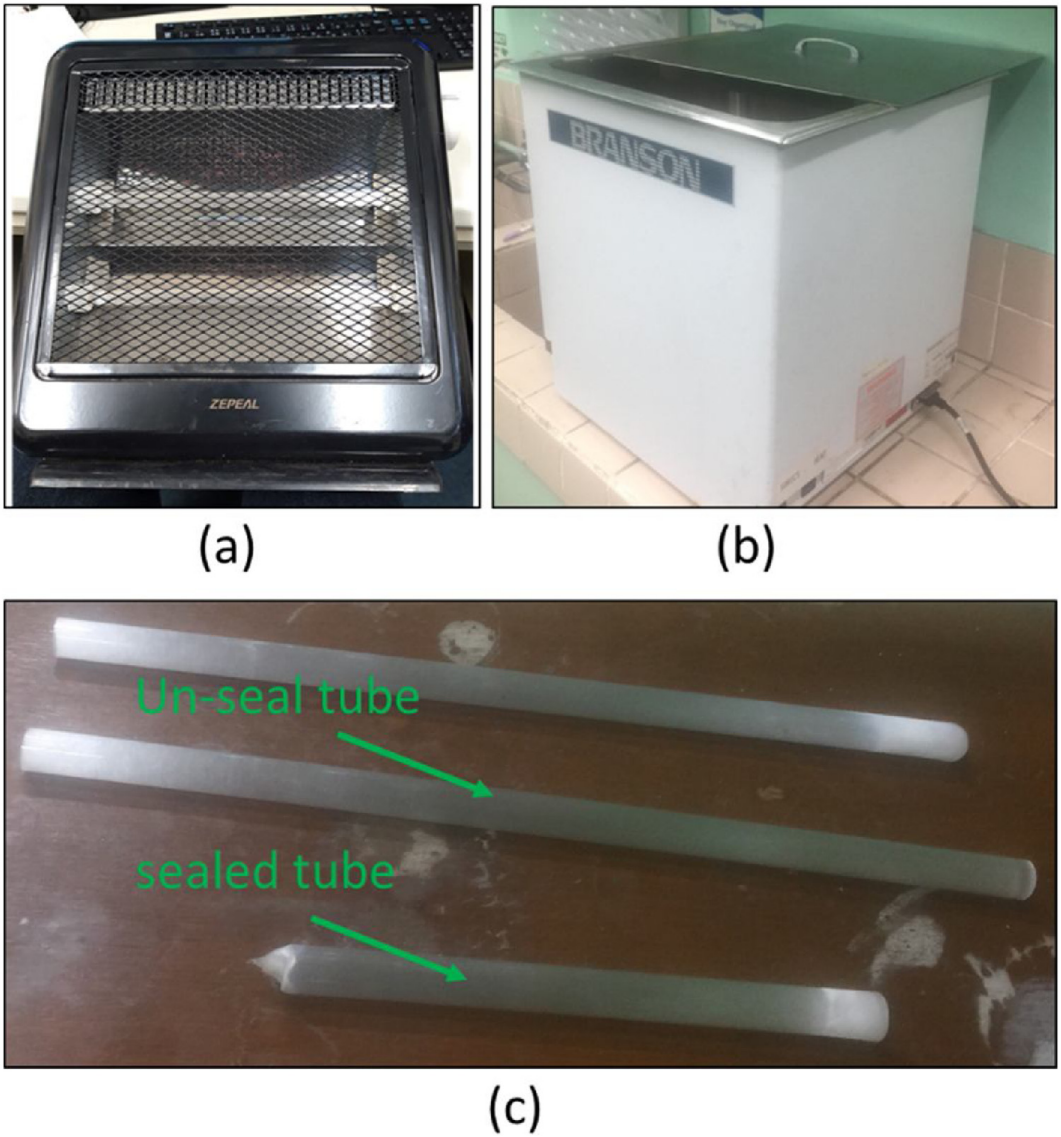
(a) Heating unit with silica tube inside, (b) ultrasonic cleaner, and (c) un-seal tube and sealed tubes.

The quartz tube was obtained from the tubes that used in toaster ovens where the main function is heating unit as shown in Fig. 3 (a). After being removed from the heating unit, the cable inside, and plastic cap at the end of the tube needs to be removed from the tube, and then placed in an ultrasonic cleaner filled with water which is ¼ of the volume of the ultrasonic cleaner (Fig. 3 (b)) to remove dirt and pollutants that might be present inside the tube. A cleaning agent can also be in the cleaning process and can be repeated several times. The tube is dried, and one-end of the tube will be sealed using a blowtorch. The tube sealed on one-end and after sealing is shown in Fig. 3 (c).

**Fig. 4:**
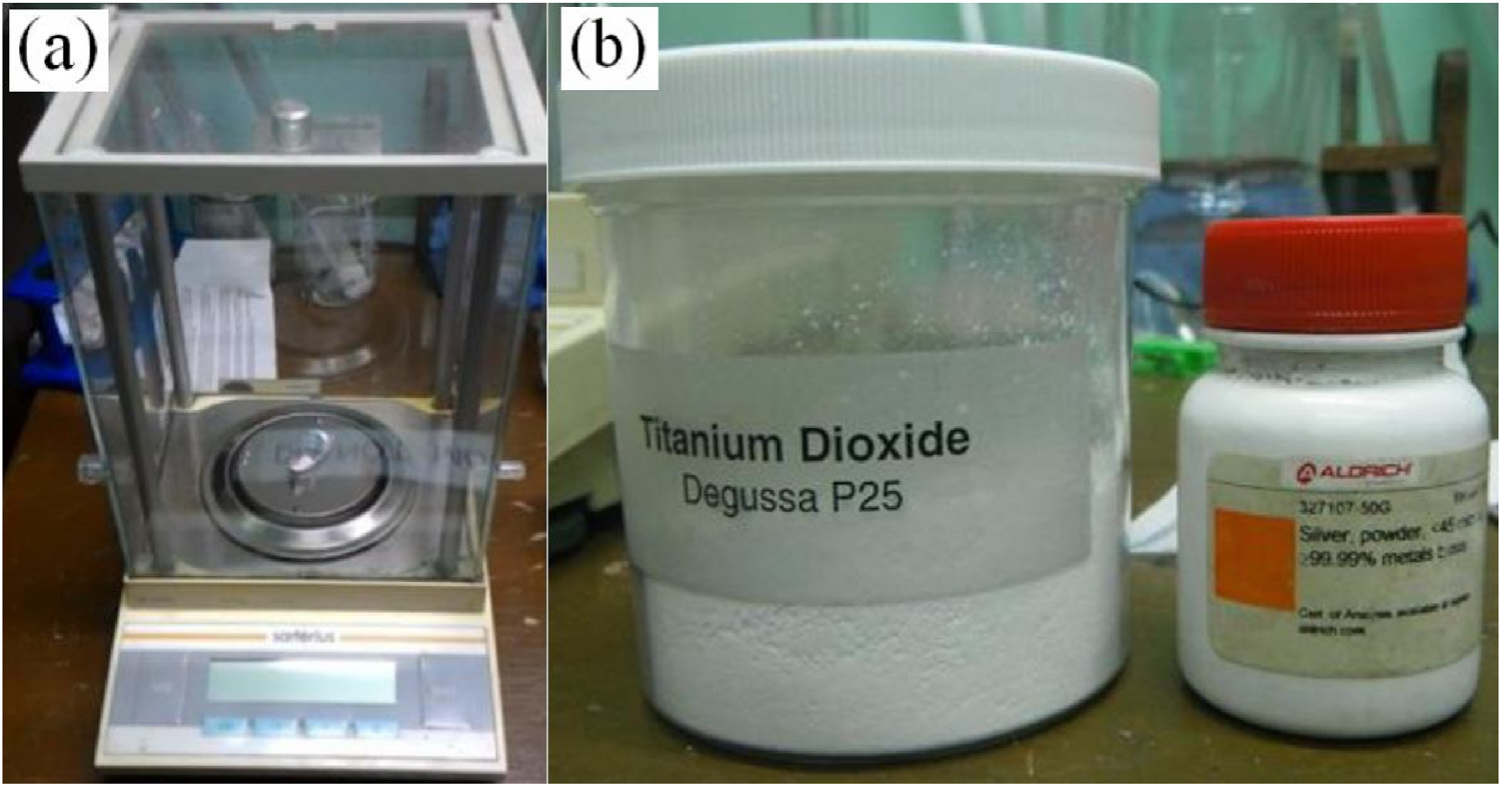
(a) digital weight scale and (b) source materials.

Before sealing, the mass of the powder material will be measured using a digital weighing scale, and then placed to the tube of one sealed end. For compound materials, the ratio can be varied based on the stoichiometric calculations. For example, a representative sample of (Ag/TiO_2_ materials), can be weighed with the amount of 17.5 mg of Ag mixed with 17.5 mg of TiO_2_. The illustration is illustrated in Fig. 4.

### Sealing process

The quartz tube that was sealed at one end and filled with powder material is then placed vertically in the Thermionic High Vacuum Machine System (THVMS). This machine is used to remove air inside the tube as well as pollutants. Before final sealing, the pressure inside the tube should reach (10^−6^ Torr ≈ 0.00013332 Pa). The vacuum pressure inside the quartz tube in essential to ensure the crystal growth of the nanomaterial based on the temperature gradient (Fig. 6) before it reaches the melting point of the material. The nanomaterial will condense in the lower temperature region of the quartz tube. The sealing process is very important since if the seal is inadequate, atmospheric pressure might enter the tube and will affect the composition of the nanomaterial.

### Note from the experiment

To ensure proper sealing, after the vacuum pressure reaches 10^−6^ Torr, let it rest in the same condition for ±30 min. When the vacuum pressure reaches a stable point, final sealing can be performed. Avoid sealing the tube if the vacuum pressure exhibits fluctuations.

### Nanomaterial growth process

In the nanomaterial growth process, the quartz tube needs to be placed horizontally inside a tube furnace. Avoid a position that is off-axis to the horizontal position. After placing the tube horizontally, place a Kaowool on both ends of the tube furnace before turning on to minimize the heat losses.

**Fig. 5:**
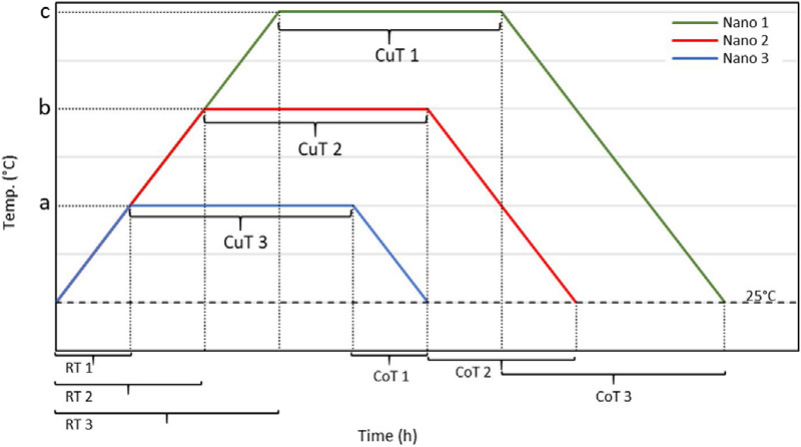
Growth graph of nanomaterial. a, b, and c related to temperature; RT is rump time (min); CuT is curing time (min.); CoT is cooling time (min.).

**Fig. 6:**
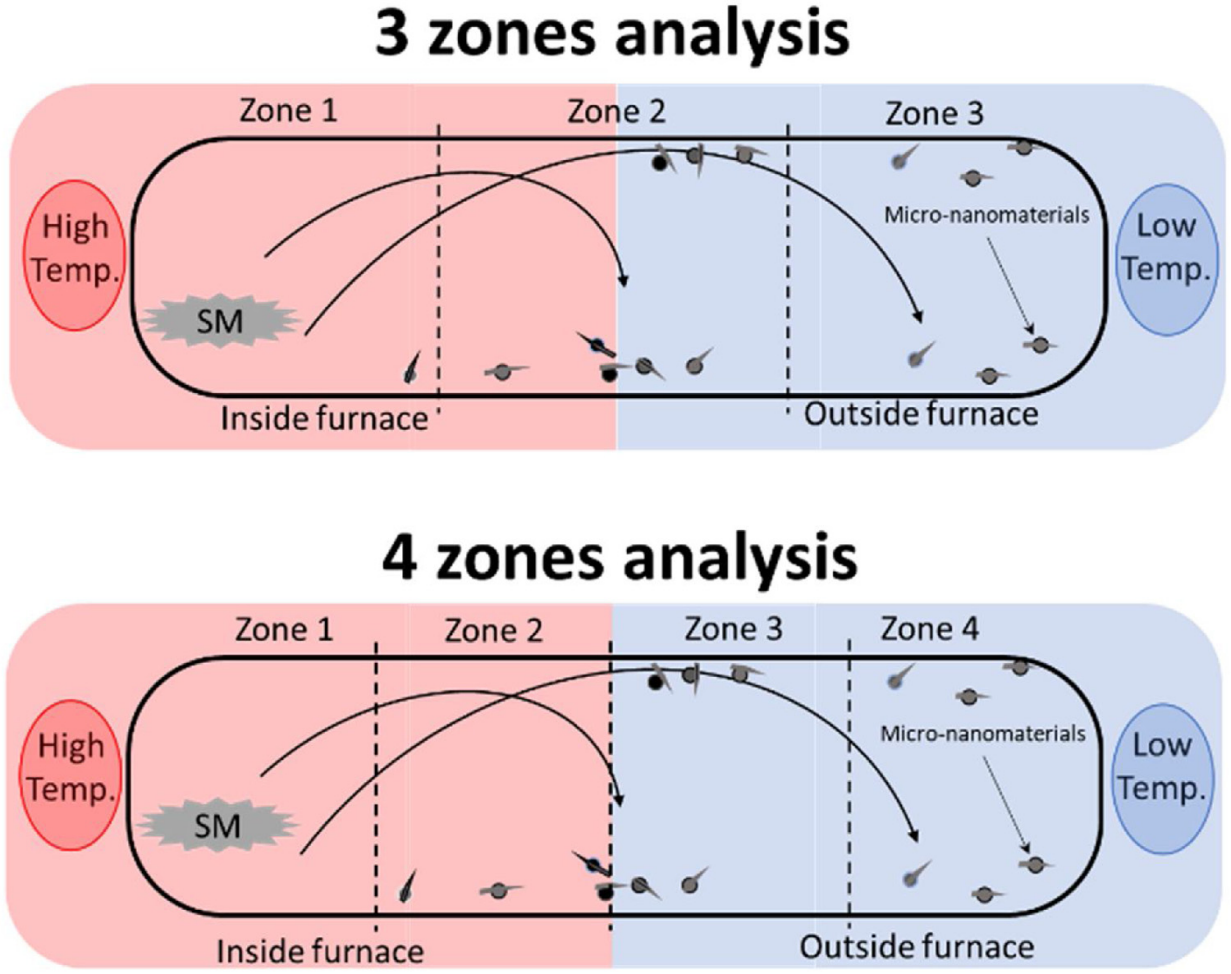
Zones difference for characterization process divided into 2 part; 3 zones and 4 zones.

The nanomaterial growth profile is shown in the Fig. 5. It begins from room temperature where the condition inside the tube is at steady state. The desired growth temperature is set with a ramp temperature 5 °C per minute. The material growth process and heat flow thru the different zones are described in Fig. 6. Generally, there are two methods to analyze nanomaterial growth using the HVPG technique; the three zone-based in region two where half of the tube is inside the furnace and the other half is exposed externally from the tube furnace, and the four zone where the region are divided into two based on temperature differences. Region 1 and Region 2 where the quartz tube is exposed to high temperature while zone 3 and zone 4 is where the condensation occur at low temperature.

**Fig. 7:**
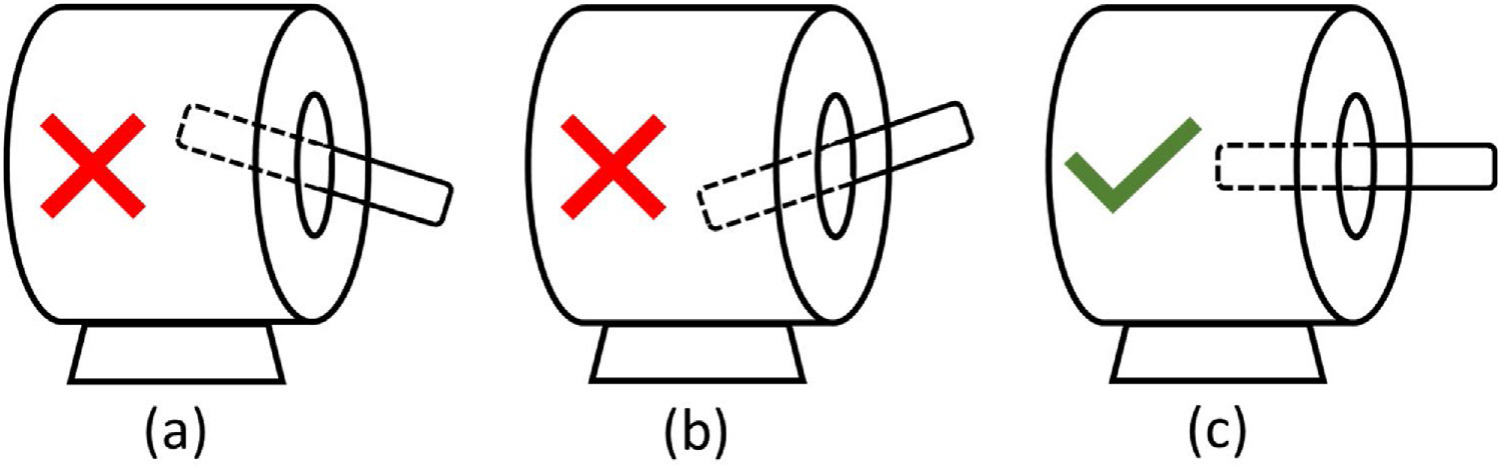
Tube position during curing process; (a) outer is risky and low growth, (b) outer has high growth with un-grown materials, and (c) horizontal is the preferred position for temperature growth.

The position of the quartz tube in the furnace is an important aspect in the nanomaterial growth process. When the position of the tube is not aligned with the horizontal, the result of the growth of the nanomaterials is not optimum. The 3 positions of the tube during growth process are shown Fig. 7. Position (a) describes the source material will grow in zone 2 or zone 3 but not in nanoscale due to low temperature and did not reach the melting point. This condition can be observed during surface characterization test where it exhibits un-growth processes mixed with the materials grown in zone 2 and zone 3. Fig. 7 (b) shows the position of the tube in zone 3 or zone 4 which is higher than the inner position (zone 1). This condition will cause the nanomaterial to be deposited in the middle and inner portions of the furnace. Position (b) has a risk that it will completely fall out of the furnace. The correct position is shown in Fig. 7 (c) where the horizontal position (in line with the furnace) is stable.

**Fig. 8:**
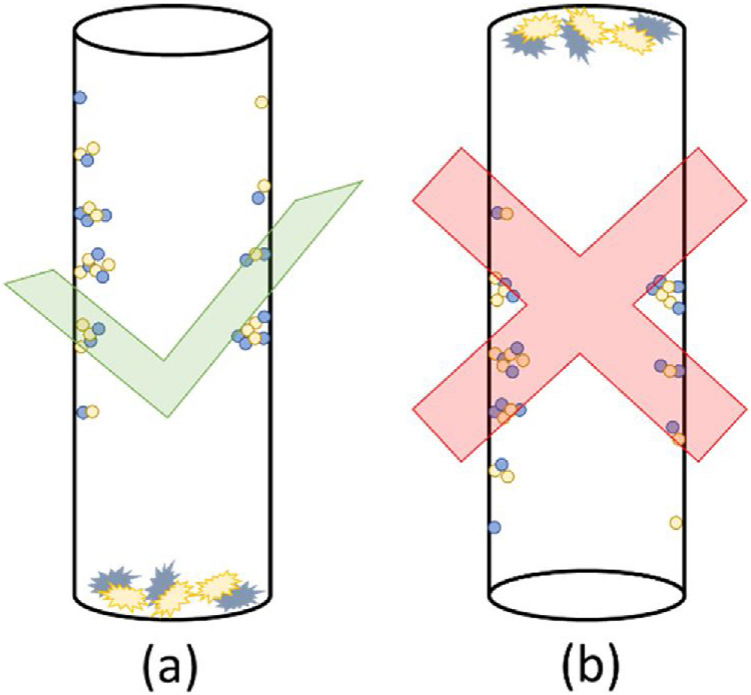
Tube position during cooling process, (a) correct position, (b) wrong position.

After the growth process is finished, the tube is then placed vertically in room temperature for 1 h to ensure the grown nanomaterial inside the tube is stable as shown in (Fig. 8 (a)). The position should not be reverse. In this procedure, if the position was inverted (Fig. 8 (b)), the material might grow to the other zones.

When the tube reaches the desired temperature of the cooling process, the tube is ready to be covered with masking tape and is slowly cracked to retrieve the nanomaterial samples. SEM is used to characterize the nanomaterial grown in each zone. At least 3 samples in each zone should be evaluated.

## Experimental results

### Synthesize Ag nanomaterial

**Fig. 9:**
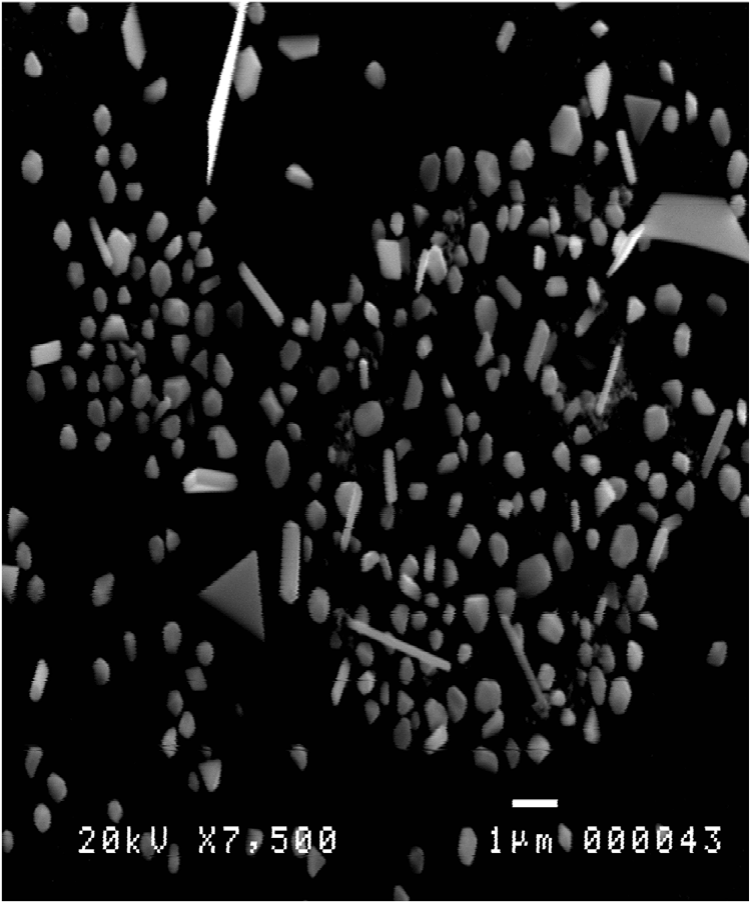
Ag nanomaterial synthesized with 8 h curing time and 1200 °C growth temperature.

Ag bulk material from Aldrich Corporation with 99 % purity was used as a source material to synthesize Ag nanomaterial using HVPG technique. It was reported that Ag nanoparticles, nanowires, triangular, and hexagonal nanomaterial were successfully grown using HVPG technique. The source materials were weighed at 35 mg in each tube and grow with different temperature parameters. The pour plate technique was used to evaluate the bactericidal property of the nanomaterials using E. coli. The study shows that Ag triangular nanomaterials can be an effective material used for anti-bacterial applications [19]. Ag nanomaterial was successfully synthesized using HVPG technique with a parameter of 8 h curing time and 1200 °C growth temperature. The result is represented in Fig. 9. It is shown that triangular nano-silver and nanoparticle are successfully synthesized.

### Synthesize Ag/Ge nanomaterial

**Fig. 10:**
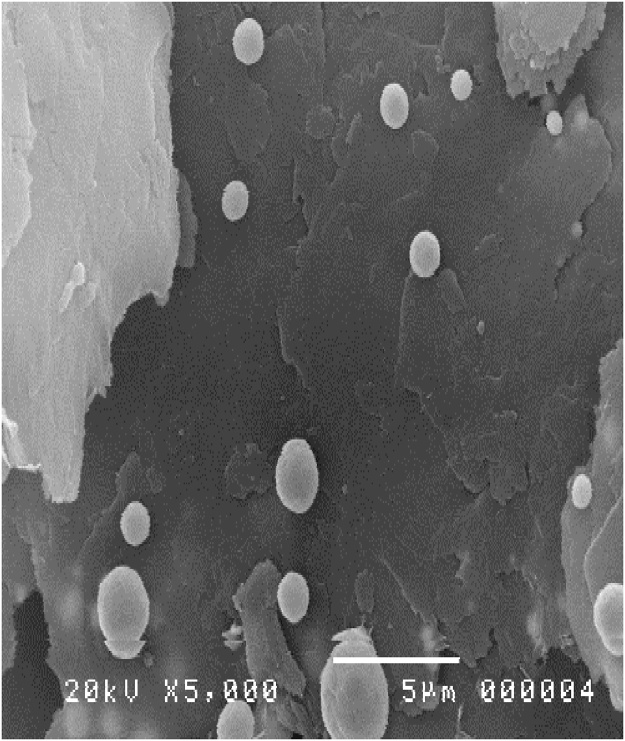
Nano silver adhered on the graphene surfaces.

HVPG technique was used to synthesize silver (Ag) powder from Aldrich combined with Graphene (Ge) multi-layer from Degussa P25. A total of 35 mg of Ag and 35 mg of Ge was added into the quartz tubes before it was sealed. The results show that silver can grow on the graphene layer with a different shape in different zones. The previous study [20] reported that nanoparticles and flower-like nanomaterial are successfully grown using HVPG technique. Further study may be used to enlarge the shape of Ag/Ge nanocomposite materials. Fig. 10 shows the silver nanoparticle adhered on the Ge multilayer. The silver sizes vary from micro-size to nano-size.

### Synthesize Ag/TiO_2_ nanomaterial

**Fig. 11:**
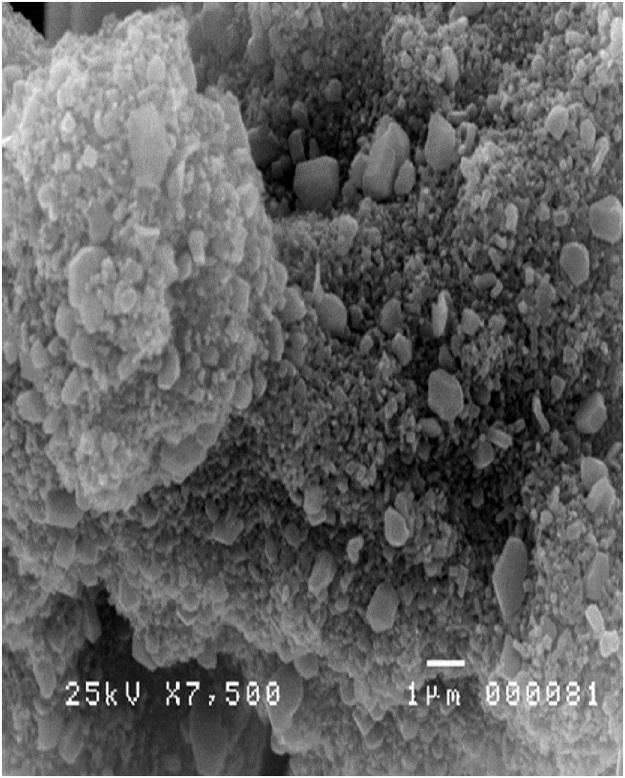
Ag/TiO_2_ Nanocomposite material.

Combination between Ag and TiO_2_ were needed to get the best combination between curing time and growth temperature because of the melting point of TiO_2_ which is higher than Ag. The composite material in the previous study shows many parameter combinations to reach the optimum. Ag micro material sourced from Aldrich while TiO_2_ was from Degussa P25. A total of 35 mg of source materials was added into the tube. The results show that by changing curing time and growth temperature, different nanomaterials were produced with different shapes, such as Nanoparticles, Nanorods, Triangular Nanomaterials, and Cotton-like Nanomaterials. The results were then evaluated using SEM-EDX to determine the best shape for different applications such as for anti-bacterial [1,21] and elemental composition. Fig. 11 shows Ag/TiO_2_ nanocomposite materials successfully synthesized using HVPG technique with a parameter of 8 h curing time and 1200 °C growth temperature.

### Synthesize SnO_2_ nanomaterial

SnO_2_ nanowires and nanorods were reported to have been successfully synthesized using HVPG technique. SnO_2_ powder provided from Merck, where 50 mg of SnO_2_ powder were loaded into the tubes. The effect of magnetic field during the synthesis process was observed. The magnetic field during the synthesis process homogenized the nanowires. The magnetic field also reported that the length of nanowires is significantly changed. Using Hydrogen Sulfide gases to evaluate SnO_2_ nanowire for meat spoilage application [22]. The study was done using 3 different curing times (4,6, and 8 h) with 1200 °C growth temperature.

### Synthesize Ag/SnO_2_ nanomaterial

Ag/SnO_2_ with different ratios (0:5, 1:4, 2:3, 3:2, 4:1, and 5:0) were successfully synthesized using HVPG technique. Ag powder was sourced from Aldrich and SnO_2_ from Merck with both of it have 99 % purity. The sample was weight at 35 mg which consists of Ag, SnO_2_, or Ag/SnO_2_ based on the designated ratio was added into the tubes. The results show that by changing parameter of curing time and growth temperature, different shapes of nanomaterials such as nanoparticles, nanowires, and cotton-like nanomaterials were grown. The study shows that Ag/SnO_2_ nanocomposite material can be used as an anti-bacterial material and coating applications [2].

**Fig. 12:**
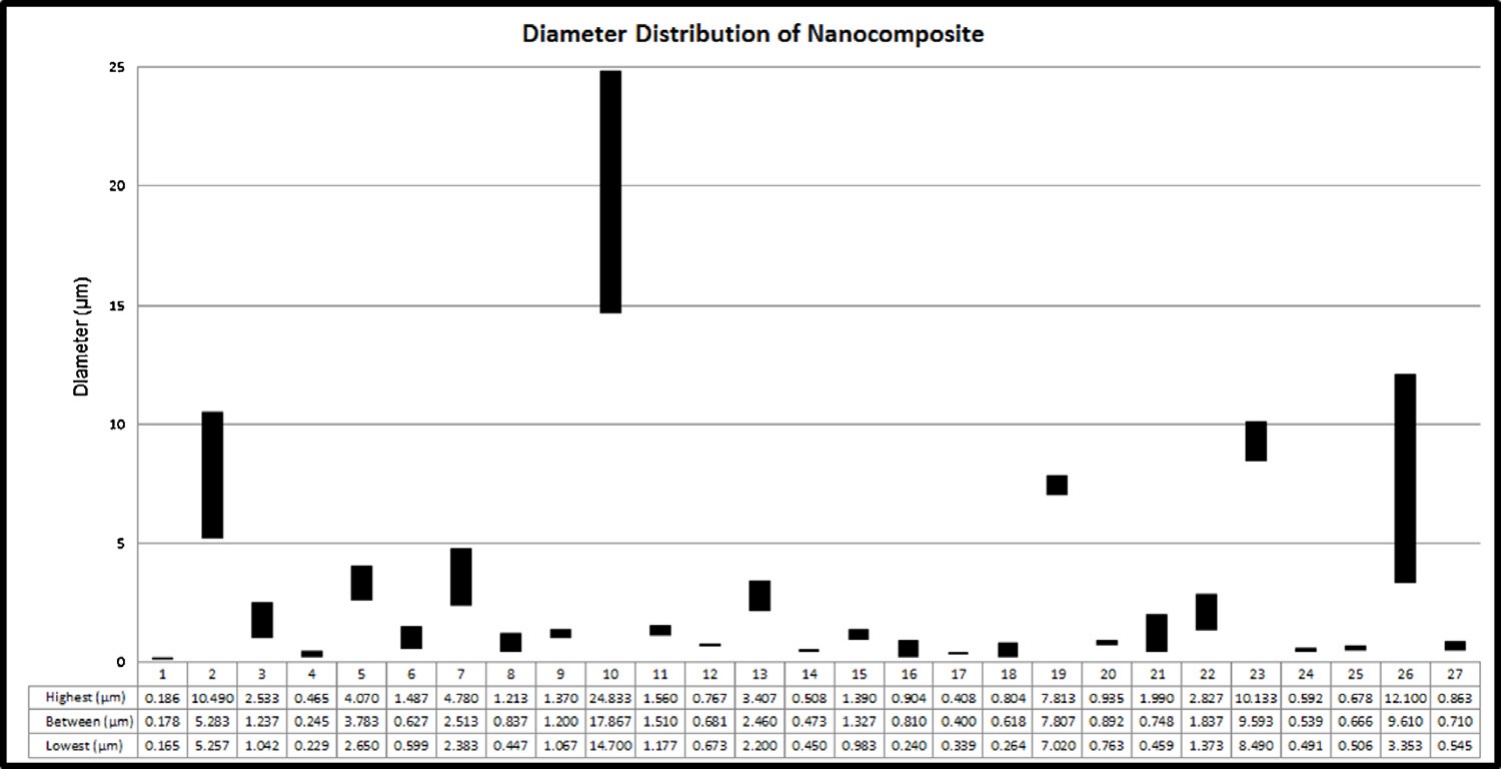
Average diameter of all specimens in the 3 zones model of Ag/TiO_2_ nanocomposite material [1].

Synthesis nanomaterial using HVPG technique contains several advantages that can be used for various applications. By changing the parameter, the nanomaterial shape can be obtained. Previous study lead by Muflikhun et al. [1] show the variation of nanomaterials synthesis from Ag/TiO_2_. The complete results of the nanomaterial shapes are shown in Table 1. The table summarizes the nanomaterial shapes in each parameter, size varying from different zones and parameters. Fig. 12 shows the average diameter of the nanomaterial in each zone, the study used Ag/TiO_2_ as representative material samples.

**Table 1:** Nanocomposite shape in all parameters (Ag/TiO_2_) [1].

No.	Temperature	Baking Time	Zone	Material Shape and diameter
1	800 °C	4 Hours	1	Nanoparticles
2			2	Microparticles
3			3	Microparticles
4		6 Hours	1	Nanospheres, Nanoparticles
5			2	Nanoparticles
6			3	Nanoparticles
7		8 Hours	1	Nanoparticles
8			2	Nanotubes, Nanospheres
9			3	Nanoparticles
10	1000 °C	4 Hours	1	Nanoparticles
11			2	Nanospheres, Nano-triangular, Nanorods
12			3	Nanospheres, Nano-triangular
13		6 Hours	1	Nanoparticles
14			2	Nanoparticles, Nanospheres
15			3	Nanoparticles
16		8 Hours	1	Nanoparticles, Nanorods
17			2	Nanorods, Nanoparticles
18			3	Nanorods, Nanoparticles
19	1200 °C	4 Hours	1	Nanoparticles
20			2	Nanospheres, Nanoparticles
21			3	Nanoparticles
22		6 Hours	1	Nanoparticles
23			2	Nanocrystal, Nano-triangular
24			3	Nanoparticles
25		8 Hours	1	Nanoparticles
26			2	Nanospheres, Nanorods, Nanocrystal
27			3	Nanorods

## Conclusion

The HVPG technique was successfully used to synthesize various nanomaterials (Ag, Ag/Ge, Ag/TiO_2_, SnO_2_, and Ag/SnO_2_) with different parameter. Ag nanomaterial was successfully synthesized with 4, 6, and 8 h curing tie followed by 800 °C, 900 °C, 1000 °C, and 1100 °C of growth temperature. Ag/Ge nanocomposite material has successfully synthesized with 1200 °C of growth temperature and 6 h baking time. Ag/TiO_2_ nanocomposite material could be synthesized with 4, 6, and 8 h curing time and 800 °C, 1000 °C, and 1200 °C of growth temperature. Synthesis SnO_2_ nanomaterial done at 1200° growth temperature and 4, 6, and 8 h of curing time. Ag/SnO_2_ was synthesized via HVPG with parameter 800 °C growth time and 6 h curing time. The different temperature during curing process creates different shapes and size of nanomaterials. The synthesis output gives a purity result up to 100 %. The results show that several nanomaterials were successfully synthesized such as Nanoparticles, Nanospheres, Nanotubes, triangular Nanomaterial, Nanorods, and Nanocrystal. The size of materials varying from 10 nm to 200 nm depends on the zones, temperature and curing time. The quartz tube is the key to this method which was used to grow different nanomaterials in the future. Combination of nanomaterial is possible, and the mass of the source material can be increased for different purposes. By using material combinations and weight ratio, the shape of the nanomaterial can be and used in various applications such as anti-bacterial, coatings, gas sensing, and other applications.

## Declaration of Competing Interest

The authors declare that they have no known competing financial interests or personal relationships that could have appeared to influence the work reported in this paper.
